# Phytochemical Compositions of Immature Wheat Bran, and Its Antioxidant Capacity, Cell Growth Inhibition, and Apoptosis Induction through Tumor Suppressor Gene

**DOI:** 10.3390/molecules21101292

**Published:** 2016-09-27

**Authors:** Mi Jeong Kim, Won-Jin Yoon, Sang Sook Kim

**Affiliations:** 1 Division of Funcitonal Food Research, Korea Food Research Institute, 62, Anyangpangyo-ro 1201 beon-gil, Bundang-gu, Seongnam-si, Gyeonggi-do 13539, Korea; Kim.Mi-jeong@kfri.re.kr; 2 Division of Strategic Food Research, Korea Food Research Institute, 62, Anyangpangyo-ro 1201 beon-gil, Bundang-gu, Seongnam-si, Gyeonggi-do 13539, Korea; Yoon.Won-jin@kfri.re.kr

**Keywords:** immature wheat bran, phytochemical composition, antioxidant capacity, cell growth inhibition, tumor suppressor genes, apoptotic cell death

## Abstract

The purpose of this study was to investigate the phytochemical compositions and antioxidant capacity, cell growth inhibition, and apoptosis induction in extracts of immature wheat bran. Immature wheat bran (IWB) was obtained from immature wheat harvested 10 days earlier than mature wheat. The phytochemical compositions of bran extract samples were analyzed by ultra-high performance liquid chromatography. The total ferulic acid (3.09 mg/g) and *p*-coumaric acid (75 µg/g) in IWB were significantly higher than in mature wheat bran (MWB, ferulic acid: 1.79 mg/g; *p*-coumaric acid: 55 µg/g). The oxygen radical absorbance capacity (ORAC: 327 µM Trolox equivalents (TE)/g) and cellular antioxidant activity (CAA: 4.59 µM Quercetin equivalents (QE)/g) of the IWB were higher than those of the MWB (ORAC: 281 µM TE/g; CAA: 0.63 µM QE/g). When assessing cell proliferation, the IWB extracts resulted in the lowest EC_50_ values against HT-29 (18.9 mg/mL), Caco-2 (7.74 mg/mL), and HeLa cells (8.17 mg/mL) among bran extract samples. Additionally, the IWB extracts increased the gene expression of p53 and PTEN (tumor suppressor genes) in HT-29 cells, indicating inhibited cell growth and induced apoptosis through tumor suppressor genes.

## 1. Introduction

Wheat (*Triticum aestivum* L.) accounts for approximately 30% of global cereal production [1] and is used as an energy source in the human diet [2]. Recently, epidemiological studies have suggested that the consumption of whole-grain products reduces the risk of heart disease [3], various types of cancer [4], and type 2 diabetes [5]. Bioactive phytochemicals, vitamins, minerals, and fiber present in the bran fraction of the grain have been reported to be responsible for most of the beneficial health effects of whole grains [6].

For these reasons, numerous studies have investigated the beneficial health effects of wheat bran in recent years [2,6,7,8]. These studies have focused on the beneficial health effects regarding the antioxidant activities and phenolic acid composition of wheat bran. Among the identified compounds in wheat bran, ferulic acid was the most abundant phenolic acid, followed by *p*-coumaric, vanillic, sinapic, syringic, and caffeic acids [8]. In addition, the phenolic compounds derived from wheat bran extract showed antiproliferative effects on cancer cells [6]. The cellular mechanisms underlying the antiproliferative activity were difficult to interpret; however, wheat bran samples potentially interfered with proliferative signal transduction among protein kinases [6]. The antioxidants in wheat bran potentially contribute to cellular defense and help prevent oxidative damage to cellular components [7]. However, the beneficial health effects of wheat bran in previous studies were limited to mature wheat bran (MWB), which is harvested in normal harvest seasons. Less information is available on the health benefits of immature wheat or its bran. 

Recently, researchers have investigated immature grains as food materials [9,10,11,12]. Immature wheat grain contains less starch and protein contents and more fiber, essential amino acids, and soluble sugars than mature wheat [10,13]. Kim and Kim [14] reported that immature wheat, compared with mature wheat, contained more phenolic and flavonoid contents and exhibited increased antioxidant and antiproliferative properties, suggesting immature wheat as a potential innovative raw material with beneficial health effects. However, no information is available regarding the individual phenolic composition of immature wheat bran or on possible mechanisms related with cell growth inhibition and apoptosis induction through tumor suppressor genes.

Therefore, the present study was aimed to investigate the phytochemical compositions and possible mechanisms related to the beneficial health effects of immature wheat or immature wheat bran (IWB). Because bioactive phytochemicals in the bran fraction are responsible for most of the health benefits in wheat, the brans from immature wheat (IW), steamed immature wheat (SIW), and mature wheat (MW) were used for this study. Steamed immature wheat was prepared to prevent spoilage or degradation by the enzymatic activity in immature wheat. First, phenolic acid compositions, antioxidant capacities, and cellular antioxidant activities of IWB and SIWB were compared with those of MWB. Second, extracts from IWB, SIWB, and MWB were examined not only for effects on cell growth inhibition and regulation of tumor suppressor genes or oncogenes of human cancer cells, but also for effects on apoptotic cell death to investigate the possible mechanisms for the anticancer properties of bran extracts. Third, correlations between the individual phenolic compositions of bran extracts and antioxidant, antiproliferative, and apoptotic cell death properties were determined by Pearson’s correlation analysis.

## 2. Results and Discussion

### 2.1. Phenolic Acid Compositions of Wheat Bran Extracts

Results of the free and bound phenolic acid compositions of the wheat bran extracts tested in this study are summarized in Table 1. Free (soluble) and bound (insoluble) fractions were extracted from each bran sample, and total phenolic acid was calculated as the sum of the free and bound phenolic acids in each sample. Phenolic acids are hydroxylated compounds which are derived from benzoic acid or cinnamic acid [15]. The results of this study indicated that hydroxycinnamic acid derivatives were more prevalent than hydroxybenzoic acid derivatives across all three samples. The hydroxycinnamic acids identified in the wheat bran samples were ferulic acid, *p*-coumaric acid, sinapic acid, and caffeic acid. The hydroxybenzoic acids identified in the three samples included syringic acid, vanillic acid, and syringaldehyde.

As shown in Table 1, free ferulic acid (the soluble fraction) significantly differed (*p *< 0.01) across the three samples, ranging from 13.37 µg/g to 26.96 µg/g (Table 1). Most (>99%) of the ferulic acid in the three samples was identified as bound ferulic acid (the insoluble fraction). The bound ferulic acids of MWB, IWB, and SIWB ranged from 1767–3071 µg/g. Although MWB (26.96 µg/g) contained more free ferulic acid than IWB (20.17 µg/g) or SIWB (13.37 µg/g), IWB (3071 µg/g) contained more bound ferulic acid than MWB (1767 µg/g) or SIWB (2532 µg/g). Ferulic acid was the predominant phenolic acid identified in the three samples, consistent with observations in previous wheat bran studies [6,16]. As reported by Kim et al. [16], total ferulic acid accounted for approximately 85% of the total phenolic acid compositions of bran samples, ranging from 1376 µg/g to 2020 µg/g. Lu et al. [6] also studied the phenolic acid compositions of ten Maryland soft winter wheat bran samples, and their results demonstrated that bound ferulic acid, as the predominant phenolic acid found in bran, ranged from 1184–1725 µg/g. In this study, the amount of bound ferulic acid in MWB (1767 µg/g) was similar to the bound ferulic acid values reported by Lu et al. [6]. However, the bound ferulic acid (3071 µg/g) identified in IWB was higher than the bound ferulic acid (1184–1725 µg/g) reported by Lu et al. [6] for soft winter wheat bran, suggesting that IWB could be more beneficial to human health than MWB.

The second most abundant phenolic acid was sinapic acid. Significant differences in free sinapic acid were also observed between MWB, IWB, and SIWB (*p *< 0.001), whereas no significant differences in bound sinapic acid were observed. In Table 1, the total sinapic acid ranged from 107–124 µg/g. This range is comparable to the total sinapic acid content (110–207 µg/g) reported by Verma et al. [8], who examined the sinapic acid content of acid- or alkaline-hydrolyzed bran of six wheat cultivars. In the present study, IWB contained more free sinapic acid (3.95 µg/g) than MWB (2.46 µg/g). These results suggested that IWB with high sinapic acid might have potential antioxidant activities. 

In addition, free and bound *p*-coumaric acid values differed significantly (both *p* < 0.001) among the three samples and were in the range of 3.22–5.61 µg/g and 51.97–74.58 µg/g, respectively. In the report of Kim et al. [16] and Verma et al. [8], *p*-coumaric acid contents in brans of various wheat cultivars were in the range of 35–46 µg/g and 133–476 µg/g, and the amount of *p*-coumaric acid varied depending on the cultivars. In the current study, the total *p*-coumaric acid content was higher than that reported by Kim et al. [16] but lower than that reported by Verma et al. [8]. In addition, the free *p*-coumaric acid (5.61 µg/g) and bound *p*-coumaric acid (68.98 µg/g) of IWB were higher than the free (3.22 µg/g) and bound *p*-coumaric acid (51.97 µg/g) of MWB. 

Significant differences in free and bound syringaldehyde contents were also observed among the three samples (both *p *< 0.001). Free syringaldehyde content ranged from 3.07 µg/g (MWB) to 12.32 µg/g (IWB), and bound syringaldehyde content ranged from 13.67 µg/g (MWB) to 18.70 µg/g (IWB). In addition, free and bound caffeic acids did not significantly differ among the three wheat bran samples. By contrast, MWB (vanillic acid: 62.52 µg/g; syringic acid: 26.11 µg/g) contained more bound vanillic acid and syringic acid than IWB (vanillic acid: 51.52 µg/g; syringic acid: 18.01 µg/g) or SIWB (vanillic acid: 47.07 µg/g; syringic acid: 19.08 µg/g). Overall, immature bran extracts contained more *p*-coumaric acid, syringaldehyde, and ferulic acid than MWB (Table 1).

### 2.2. Antioxidant Capacity of Bran Extract Samples

Antioxidant capacity is frequently used as an important index for health benefits in foods, and several methods are used to determine in vitro antioxidant properties [17]. In this study, the antioxidant capacities of bran samples were measured using oxygen radical absorbance capacity (ORAC) and cellular antioxidant activity (CAA). The results of antioxidant capacities are in Table 2.

The free ORAC value significantly differed among the three samples (*p *< 0.01) and ranged from 38–54 µM TE/g. Significant differences in bound ORAC values were also observed among the three samples (*p* < 0.001). Recently, Lu et al. [6] studied the antioxidant activities of bran fractions from ten Maryland-grown soft winter wheat cultivars and reported that the ORAC values of the wheat bran samples were in the range of 39.91–61.50 µM TE/g. The free ORAC values (38–54 µM TE/g) in the current study are comparable to the ORAC values reported by Lu et al. [6]. According to Lu et al. [6], 50% acetone was used for the extraction of phenolics to measure antioxidant activities, and only free phenolic fractions were extracted. In this study, the bound ORAC value was six times higher than the free ORAC values, indicating significant contributions to ORAC by the bound phenolics in bran. As reported by Hung [18], the phenolic acids of wheat exist mostly in the bound form. Bound phenolics are considered to possess more health benefits than free phenolics because bound phenolics in wheat appear to serve as powerful antioxidants by radical scavenging. In the present study, the highest bound ORAC values were observed in the bran extract of the IWB (273 µM TE/g), followed by SIWB (252 µM TE/g) and MWB (237 µM TE/g). These results suggest that the health benefits from IWB might be stronger than those from SIWB or MWB.

Moore et al. [19] reported that the total ORAC values of eight whole-wheat samples ranged from 32.9 µM TE/g to 47.7 µM TE/g, which were similar to free ORAC values (38–54 µM TE/g) in this study. Total ORAC values (281–327 µM TE/g) in this study were much higher than those reported by Moore et al. [19]. Additionally, the free ORAC values (19.6–37.5 µM TE/g) of whole wheat reported by Okarter et al. [20] was comparable to the free ORAC values of the current study, whereas the bound ORAC values (31.9–59.5 µM TE/g) reported by Okarter et al. [20] were lower than the bound ORAC values (281–327 µM TE/g) of bran reported in this study. These results indicate that the bran fraction of wheat contains more antioxidant capacity than whole wheat, and the antioxidant capacity in the bound fractions of bran is higher than in the free fractions. Because phenolic compounds of wheat are concentrated in the outermost layers, brans obtained from milling may be used as a natural source of antioxidants [21]. The results of this study suggest wheat bran is a functional food ingredient that may exert positive health effects [22]. In addition, results of this study demonstrate that IWB exhibits higher antioxidant activity than MWB.

The CAA assay can evaluate the cellular-based antioxidant activity of foods more accurately than chemical methods [23]. The CAA assay is more physiologically relevant to biological systems, which are complex in nature and different from chemical systems [17]. In this study, the cellular antioxidant activities of the bran extracts were measured on HepG2 cells and expressed as µM QE/g of bran. Significant differences (*p *< 0.001) in the cellular antioxidant activities of the bran extracts were observed among the three bran samples. IWB had the highest CAA value (4.59 µM QE/g), whereas MWB exhibited the lowest value, 0.63 µM QE/g (Table 2). The CAA value measured in the IWB extract was higher than the reported levels from other grains [17,24,25], suggesting that IWB can be utilized as an effective ingredient in functional foods to help prevent various cancers or chronic diseases.

### 2.3. Effect of IWB Extract on Cell Growth of Human Carcinoma Cells

**Figure 1:**
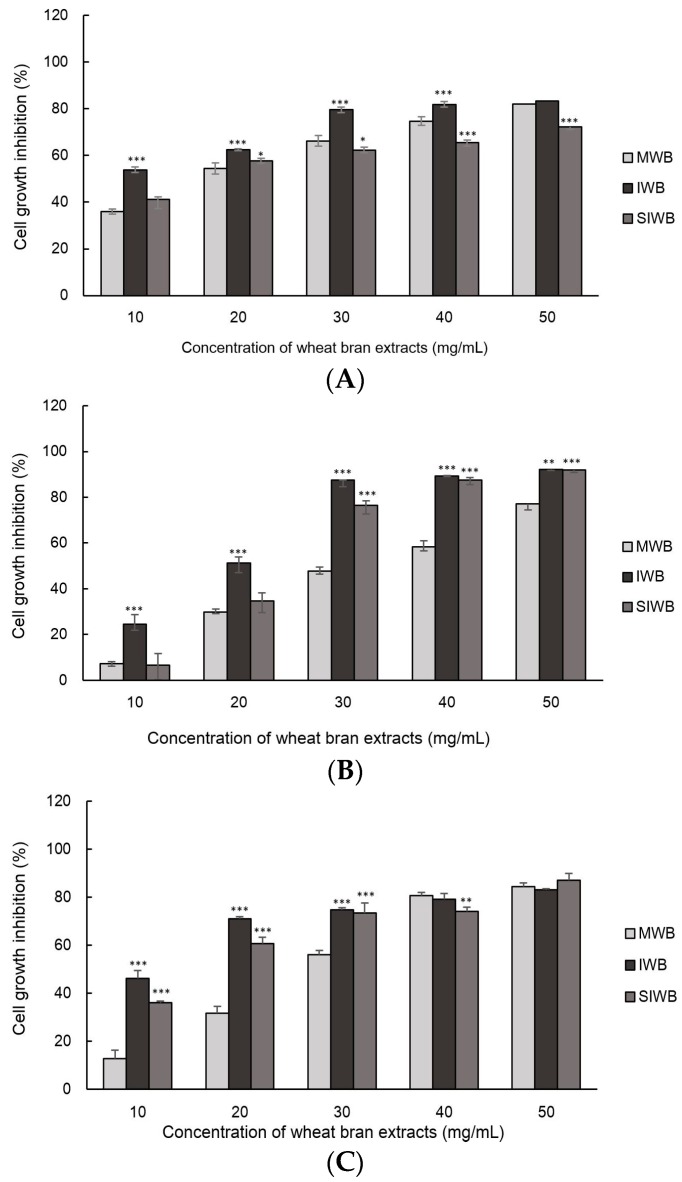
The cell growth inhibition of (**A**) Caco-2; (**B**) HT-29; and (**C**) HeLa cells treated with mature wheat bran (MWB), immature wheat bran (IWB), and steamed immature wheat bran (SIWB) extracts. * *p *< 0.05, ** *p *< 0.01, *** *p *< 0.001 vs. MWB at each concentration of wheat bran extracts.

The cell growth inhibition of Caco-2, HT-29, and HeLa cells treated with wheat bran extracts are illustrated in Figure 1. Differences in cell growth inhibition of HT-29, Caco-2, and HeLa cells were observed among the three bran samples (*p *< 0.001). 

In this study, IWB exhibited the highest inhibition of Caco-2, HT-29, and HeLa cells. For example, 30 mg/mL of IWB extract inhibited approximately 80%, 87%, and 75% of Caco-2, HT-29, and HeLa cell proliferation, respectively. By contrast, 30 mg/mL of MWB extract exhibited the lowest inhibition (66%, 48%, and 56% for Caco-2, HT-29, and HeLa cells, respectively). At 30 mg/mL of bran extract, IWB extract resulted in a two-fold reduction in proliferation of HT-29 cells compared with the MWB extract.

The median effective dose (EC_50_) of the three wheat bran extracts for antiproliferative activity against Caco-2, HT-29, and HeLa cells are shown in Table 3. Lower EC_50_ values indicate higher inhibition of cell proliferation. Among the three wheat bran extract samples, MWB exhibited the lowest inhibition of cell proliferation in Caco-2, HT-29, and HeLa cells. The EC_50_ of IWB for Caco-2 cells was 7.62 mg/mL, whereas those of SIWB and MWB were 17.25 mg/mL and 15.62 mg/mL, respectively, demonstrating increased inhibition of Caco-2 cells by IWB compared with SIWB and MWB. Overall, the EC_50_ values were the highest for HT-29 cells, indicating relatively high proliferative activities in these cells.

Previous studies have reported that cereals with antioxidant activities have cancer-protective effects [5,17,25], suggesting that natural antioxidants from cereals can inhibit cancer cell growth. In this study, the IWB exhibited increased antioxidant capacity and antiproliferative activity compared with the other samples, suggesting potential for IWB as a functional food ingredient with antioxidant capacity and anticancer effects.

### 2.4. Induction of Apoptosis by Bran Extract

**Figure 2:**
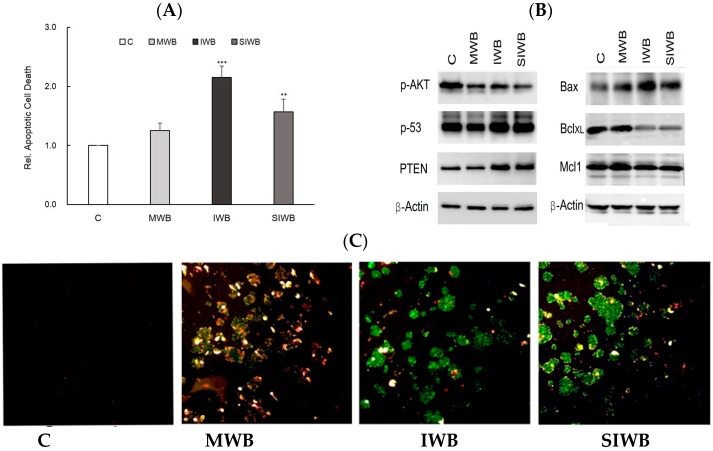
Apoptotic cell death and regulation of tumor suppressor gene or oncogene of HT-29 cells treated with mature wheat bran (MWB), immature wheat bran (IWB), and steamed immature wheat bran (SIWB) extracts. (**A**) depicts relative apoptotic cell death results measured with a Cell Death Detection enzyme-linked immunosorbent assay (ELISA) kit; (**B**) shows Western blotting results; (**C**) represents observation of cells stained with Annexin V and propidium iodide (PI) by fluorescent microscopy with 40× magnification. ** *p *< 0.01, *** *p *< 0.001 vs. C (C, cells without treatment).

The levels of apoptotic cell death of HT-29 cells treated with the three bran extracts are presented in Figure 2. Wang et al. [24] reported that grain extracts exhibited antiproliferative properties in human cancer cells, and the mechanism was associated with human cancer cell apoptosis. To investigate whether bran extracts could induce apoptosis in HT-29 cells, relative apoptotic cell death was measured using a Cell Death Detection enzyme-linked immunosorbent assay (ELISA) kit, and the results are presented in Figure 2A. As indicated in Figure 2A, the IWB extract induced more apoptotic cell death than did the MWB extract. PTEN functions as a tumor suppressor by negatively regulating the AKT/PKB signaling pathway [26]. As a general mediator of survival signals, AKT/PKB is an important upstream negative regulator of p53 [26]. Thus, pAKT might play a critical role in controlling survival and apoptosis. As shown in Figure 2B, gene expression of p53 and PTEN increased in HT-29 cells when treated with IWB or SIWB extracts. By contrast, gene expression of pAKT decreased in HT-29 cells when treated with IWB or SIWB extracts, suggesting that IWB and SIWB extracts inhibit the survival of HT-29 cells. In addition, apoptotic cell properties induced by IWB and SIWB extracts were observed by increasing gene expression of Bax and decreasing gene expression of Bcl-XL and Mcl-1 (Figure 2B).

Previous studies have reported that Bcl-XL and Mcl-1, among the anti-apoptosis Bcl-2 family members, promoted cell survival by inhibiting apoptotic activity [27]. However, Bax, from the pro-apoptosis Bcl-2 family, contributed to mitochondrial-mediated apoptosis, which is involved in the release of mitochondrial cytochrome c into the cytoplasm [28]. These results were also confirmed by observations with Annexin V and propidium iodide (PI) staining after treatment for 24 h with 10 mg/mL bran extracts via fluorescent microscopy. A representative image of bran extract-treated and untreated HT-29 cells is provided in Figure 2C. The untreated cells did not exhibit any staining, suggesting that they did not undergo significant apoptosis or necrosis. In Figure 2C, green and red stained cells indicate apoptotic and necrotic cells, respectively. Yellow-stained cells represent late apoptotic cells. As shown in Figure 2C, more green cells were observed after treatment with the IWB extract than after treatment with the MWB extract, indicating that the IWB extract effectively induced apoptosis.

### 2.5. Correlations between Phenolic Compositions and Antioxidant, Antiprolferative, and Apoptosis Cell Death Properties in Wheat Bran Samples

The correlation coefficients (r) between phenolic compositions and antioxidant, antiproliferative, and apoptotic cell death properties among the three wheat bran samples are summarized in Table 4. ORAC values were correlated with bound ferulic acid (r = 0.815), free *p*-coumaric acid (r = 0.823), bound *p*-coumaric acid (r = 0.779), free syringaldehyde (r = 0.713), and bound syringaldehyde (r = 0.781). A strong correlation was observed between CAA values and bound ferulic acid (r = 0.988), free sinapic acid (r = 0.914), free *p*-coumaric acid (r = 0.961), bound *p*-coumaric acid (r = 0.950), free syringaldehyde (r = 0.940), and bound syringaldehyde (r = 0.961). Several in vitro and in vivo studies have demonstrated that phenolic acids function as antioxidants and the antioxidant properties of phenolic acids are mainly attributed to electron donation and hydrogen atom transfer to free radicals [7,29]. 

As shown in Table 4, bound ferulic acid, free sinapic acid, *p*-coumaric acid, and syringaldehyde exhibited high correlations with the antioxidant properties of wheat bran extracts. Dai and Mumper [30] suggested that a diet including a high consumption of antioxidant-rich foods significantly reduces the risk of many cancers. In this study, strong correlations were identified between antiproliferative activities against HT-29, Caco-2, and HeLa cells and bound ferulic acid (r = 0.863 in HT-29 cells; r = 0.939 in Caco-2 cells; r = 0.975 in HeLa cells) and free sinapic acid (r = 0.786 in HT-29 cells; r = 0.826 in Caco-2 cells; r = 0.927 in HeLa cells). In addition, antiproliferative activities against cancer cells used in this study were correlated with *p*-coumaric acid and syringaldehyde. Apoptotic cell death was correlated with bound ferulic acid (r = 0.882), free sinapic acid (r = 0.828), free *p*-coumaric acid (r = 0.795), bound *p*-coumaric acid (r = 0.966), free syringaldehyde (r = 0.812), and bound syringaldehyde (r = 0.890). As indicated in Table 1, IWB contained more bound ferulic acid, free sinapic acid, *p*-coumaric acid, and syringaldehyde than MWB, suggesting effective antioxidant and anticancer properties in IWB. Overall, the results of this study demonstrated that IWB exhibited high antioxidant and anticancer properties, implying potential health benefits of IWB.

## 3. Materials and Methods 

### 3.1. Wheat Bran Samples

The wheat bran samples were gained by milling of wheat kernels. The wheat (*Triticum aestivum* L. cv. Keumkang) used in this study was grown in Iksan (Jeollabuk-Do, Korea) and harvested in 2014. Immature wheat was harvested 10 days earlier than mature wheat, which was harvested 45 days after the heading date. In addition, immature wheat was steamed for 30 s with 0.5 Mpa at 100 °C to obtain steamed immature wheat. The mature, immature, and steamed immature wheat were dried overnight using a dry oven (HK-D0100F, Hankuk General Equipment Plant, Hwaseong-si, Korea). Before milling, wheat kernels were tempered overnight to 16% moisture content and milled using a Bühler experimental mill (Bühler, Uzwil, Switzerland) at a 60% extraction rate. The bran fractions obtained from three samples were in the range of 15.0%–16.0% (*w*/*w*) of wheat kernels. Each bran sample was ground into powder using a Cyclotec™ 1903 sample mill (Foss, Hillerod, Denmark) and stored at 4 °C for further analysis.

### 3.2. Chemicals and Reagents

Trolox, quercetin, fluorescein (FL), dimethyl sulfoxide (DMSO), 2′,7′-dichlorofluorescin diacetate (DCFH-DA), vanillic acid, caffeic acid, syringic acid, *p*-coumaric acid, syringaldehyde, ferulic acid, and sinapic acid were purchased from Sigma-Aldrich, Inc. (St. Louis, MO, USA). Monopotassium phosphate, dipotassium phosphate, ethanol, methanol, hydrochloric acid, sodium hydroxide, hexane, acetic acid, acetonitrile, and water were obtained from Junsei Chemical Co., Ltd. (Tokyo, Japan), and 2,2′-azobis(2-amidinoprpane) dihydrochloride solution (ABAP) was purchased from Wako Chemicals Inc. (Richmond, VA, USA). HT-29, Caco-2, HeLa, and HepG2 cells were obtained from the American Type Culture Collection (ATCC) (Manassas, VA, USA). McCoy’s 5A, MEM, fetal bovine serum (FBS), phosphate-buffered saline (PBS), and Trypsin-EDTA were purchased from Welgene (Daegu, Korea). Dulbecco’s Modified Eagle Medium (DMEM) was purchased from HyClone Laboratories Inc., (South Logan, UT, USA). A MTT cell proliferation assay kit and a Cell Death Detection ELISA^plus^ kit were purchased from Roche Ltd., (Indianapolis, IN, USA). The p-AKT, p-53, PTEN, β-Actin, Bax, Bcl-XL, and Mcl-1 were obtained from Cell Signaling Technology (Beverly, MA, USA). ECL prime western blotting detection reagent was purchased from GE Healthcare Life Sciences (Marlborough, MA, USA).

### 3.3. Preparation of Free and Bound Extracts from Wheat Bran

MWB, IWB, and SIWB were extracted according to the procedure from Kim and Kim [14]. For the soluble (free fraction) bran extracts, 1 g of each sample was extracted with 20 mL of ethanol/water 80/20 (*v*/*v*) for 10 min. The supernatant was collected in a flask, and the extraction procedure was repeated. The collected supernatants were evaporated to dryness at 45 °C, and the dry samples were redissolved in 5 mL of methanol/hydrochloric acid 80/20 (*v*/*v*).

To obtain the bound fractions, the residues from the free fraction were mixed with 6 M sodium hydroxide for 1 h and then washed with hexane to remove lipids. The mixture was next hydrolyzed with hydrochloric acid at 85 °C for 30 min and then extracted with ethyl acetate. The collected supernatants were evaporated to dryness at 45 °C and redissolved in 10 mL of methanol/hydrochloric acid 80/20 (*v*/*v*). The extracts were used to measure phenolic acid compositions and oxygen radical absorbance capacity (ORAC).

### 3.4. Determination of Phenolic Compounds by Ultra-High Performance Liquid Chromatography

The phenolic compounds of the wheat bran extracts were analyzed as described by Okarter et al. [20]. The separation of individual phenolic compounds was performed using *u*-HPLC system (LaChromUltra L-2000 U-series apparatus, Hitachi-high Technologies Crop, Tokyo, Japan) consisting of a pump (Hitachi L-2160U, Tokyo, Japan), an autoinjector (Hitachi L-2200U, Tokyo, Japan), and an array detector (Hitachi L-2455U Diode array detector, Tokyo, Japan). The chromatography was performed using a C18 column (2 µm, 100 mm × 2.0 mm I.D, Hitachi LaChromUltra C18, Tokyo, Japan). Two solvent [water adjusted to pH 2.8 with acetic acid (A) and acetonitrile/water 70:30 *v*/*v* adjusted to pH 2.8 with acetic acid (B)] were used as the mobile phase and delivered at a flow rate of 1.5 mL/min with gradient program as described in Okarter et al. [20]. The total run time was 20 min with a 6 min delay between injections. Twenty microliters of sample were injected and each injection was monitored at 282 nm. The identification of each peak was confirmed using the retention time and absorbance spectrum of each pure compound. Data signals were acquired and processed using EZChrome Elite software (Hitachi, Tokyo, Japan).

### 3.5. Determination of Antioxidant Capacity

Hydrophilic antioxidant capacity was determined using an oxygen radical absorbance capacity (ORAC) assay described by Lu et al. [6] with modification of the sample amounts. The bran extracts were diluted with 75 mM phosphate buffer (pH 7.4), and FL was used as the fluorescent probe. In brief, 20 μL of diluted extracts (as antioxidants), 75 mM phosphate buffer, or Trolox (as a standard calibration solution at 6.25–50 μM), and 200 μL FL were transferred to 96-well black plates. After incubation for 30 min at 37 °C, 20 μL of 79.6 μM ABAP (as a peroxyl radical generator) was added to each well. FL intensity was measured using a SpectraMax^®^ i3 plate reader (Molecular Devices, Sunnyvale, CA, USA) at an excitation wavelength of 485 nm and an emission wavelength of 520 nm. The results were expressed as μM Trolox equivalents (TE) per gram of wheat bran. The total ORAC value of each sample was calculated by summing the free and bound ORAC values.

### 3.6. Preparation of Bran Nextract for Treatment in Cell Cultures

Approximately 10 g of wheat bran was extracted in 80% chilled ethanol, evaporated to dryness at 45 °C, and re-dissolved in DMSO as previously described by Whent et al. [31]. In this study, HT-29, Caco-2, HeLa, and HepG2 cells were used to examine cellular antioxidant activity, cell growth, and apoptotic cell death. The cells were supplemented with 10 or 20% FBS and grown at 37 °C in a humidified incubator with 5% CO_2_.

### 3.7. Determination of Cellular Antioxidant Activity

In vivo antioxidant capacities of the wheat bran samples were measured by cellular antioxidant activity (CAA) described by Wolfe and Liu (2007) [32]. First, the HepG2 cells (6 × 10^4^ cells/well) were seeded in black-walled 96-well plates for 24 h. After they were incubated overnight, the cells were washed with PBS and then treated with medium including bran extracts (as antioxidants) or quercetin (as standards) and 25 µM DCFHDA. After 1 h, 100 µL of 600 µM ABAP was added into each well. Fluorescence was read using a SpectraMax^®^ i3 plate reader (Molecular Devices, Sunnyvale, CA, USA) at an excitation wavelength of 485 nm and an emission wavelength of 538 nm at 5 min intervals for 1 h. The CAA value of each extract was expressed as µM quercetin equivalents per gram of bran samples.

### 3.8. Determination of Cell Growth and Apoptotic Cell Death

HT-29, Caco-2, and HeLa cells (1 × 10^4^) were seeded in 96-well plates for 24 h. Then, the cells were treated with various concentrations of the extracts (0–50 mg/mL) for 72 h at 37 °C in a humidified incubator with 5% CO_2_. After 72 h of incubation, cell growth was determined using a MTT Cell Proliferation Assay kit with a SpectraMax^®^ i3 plate reader at 570–655 nm (Molecular Devices, Sunnyvale, CA, USA).

To measure apoptotic cell death, HT-29 cells (1.5 × 10^5^) were incubated overnight. The medium was replaced with medium containing 10 mg/mL bran extracts, and then the cells were incubated for 48 h at 37 °C in a humidified incubator with 5% CO_2_. After incubation, relative apoptotic cell death was measured using a Cell Death Detection ELISA^plus^ kit with a SpectraMax^®^ i3 plate reader (Molecular Devices) at a test wavelength of 405 nm and a reference wavelength of 490 nm.

### 3.9. Western Immunoblots

Protein expression was detected by western blotting, as previously described by Ferguson et al. [33]. In brief, cells were harvested with a scraper and then washed with cold PBS twice. The cells were then lysed in radio immunoprecipitation assay (RIPA) buffer containing protease inhibitors. Following cell lysis by sonication and centrifugation at 14,000× *g* for 15 min at 4 °C, the resulting supernatant was collected as the total cell lysate. As previously described, western blotting was performed by separating 50 µg of protein per lane via 10 to 12% sodium dodecyl sulfate polyacrylamide gel electrophoresis (SDS-PAGE) followed by transfer of the proteins to polyvinylidene difluoride (PVDF) membranes. Membranes were blocked with 5% skim milk in Tris-buffered saline containing Tween 20 (TBST) for 1 h at room temperature, and then incubated with primary antibodies (p-AKT, p-53, PTEN, β-Actin, Bax, Bcl-XL, and Mcl-1) at 4 °C. After overnight incubation, the membranes were washed and then incubated with secondary antibody at room temperature for 1 h. Next, the membranes were developed using ECL prime western blotting detection reagent.

### 3.10. Apopttic Cell Morphology by Green Fluorescent Protein

HT-29 cells were seeded into four chamber slide (SPL, Gyeonggi-do, Korea) at a density of 1 × 10^6^ cells/chamber. The attached cells were then treated with 10 mg/mL bran extracts for 24 h, and then the slides were washed with PBS. After the chambers were washed, each slide received 50 µL of Dual Detection Reagent containing apoptosis detection reagent (Green Fluorescent Protein/Fluorescein isothiocyanate, GFP/FITC) and necrosis detection reagent (7-AAD) in 1× binding buffer. The samples were incubated at room temperature for 15 min under dark conditions. After staining, the cells were washed with binding buffer and covered with a glass coverslip. The stained cells were observed using a fluorescence microscope (Olympus IX71) at a magnification of 40× with a filter set for detecting the apoptosis (Excitation/Emission wavelength: 550/570 nm) and necrosis reagents (Excitation/Emission wavelength: 546/647 nm).

### 3.11. Statistical Analysis

Three replicates of experiments were performed, and all data are presented as the mean ± standard deviation. An analysis of variance (ANOVA) and a Student Newman-Keuls (SNK) multiple comparison were performed using Statistical Analysis System ver. 9.2 (SAS Institute, Cary, NC, USA) to identify differences between samples. Pearson’s correlation analysis was performed to determine the relationships between the various phenolic acid compositions and antioxidant capacities, cellular antioxidant activities, cell proliferation, and apoptotic cell death of the three wheat bran samples.

## 4. Conclusions 

Wheat bran is generally considered a by-product of the flour milling industry, and its phenolic acids are sources of antioxidants that inhibit various diseases, including cancer. The use of IWB as a functional ingredient remains limited because of a lack of information regarding the advantages of IWB. Recently, researchers have begun to focus on the functionality of ingredients conveying health benefits. The present study indicated that IWB contained more phenolic acids and exhibited higher antioxidant properties than MWB. Moreover, IWB extracts inhibited the proliferation of cancer cells and induced apoptosis more strongly than MWB extracts. As the first report on the cellular antioxidant capacity and activation of tumor suppressor genes related to the beneficial health effects of IWB, the results of this study could provide a foundation for future research on the health benefits of IWB and its utilization.

## Figures and Tables

**Table 1:** Phenolic acid compositions in extracts of mature wheat bran (MWB), immature wheat bran (IWB), and steamed immature wheat brans (SIWB).

Phenolic Acid Composition ^1^		MWB	IWB	SIWB
Ferulic acid (µg/g)	Free ***	26.96 ± 2.78 ^a^	20.17 ± 0.38 ^b^	13.37 ± 0.47 ^c^
	Bound ***	1767 ± 3.00 ^c^	3071 ± 32.8 ^a^	2532 ± 45.7 ^b^
	Total ^2,^***	1794 ± 2.80 ^c^	3091 ± 32.9 ^a^	2545 ± 45.4 ^b^
Sinapic acid (µg/g)	Free ***	2.46 ± 0.18 ^b^	3.95 ± 0.44 ^a^	3.51 ± 0.22 ^a^
	Bound	118 ± 1.68 ^a^	103 ± 21.12 ^a^	120 ± 3.00 ^a^
	Total ^2^	121 ± 1.79 ^a^	107 ± 21.34 ^a^	124 ± 2.90 ^a^
Vanillic acid (µg/g)	Free **	11.98 ± 0.21 ^a^	12.20 ± 1.95 ^a^	8.07 ± 0.52 ^b^
	Bound ***	62.52 ± 0.21 ^a^	51.52 ± 1.31 ^b^	47.07 ± 1.61 ^c^
	Total ^2,^***	74.49 ± 0.24 ^a^	63.73 ± 1.70 ^b^	55.14 ± 1.41 ^c^
*p*-Coumaric acid (µg/g)	Free ***	3.22 ± 0.07 ^c^	5.61 ± 0.32 ^a^	4.68 ± 0.51 ^b^
	Bound ***	51.97 ± 1.60 ^c^	68.98 ± 1.51 ^a^	58.93 ± 1.75 ^b^
	Total ^2,^***	55.19 ± 1.65 ^c^	74.58 ± 1.38 ^a^	63.61 ± 1.53 ^b^
Syringic acid (µg/g)	Free	3.38 ± 0.03 ^a^	3.63 ± 0.03 ^a^	2.39 ± 0.31 ^a^
	Bound **	26.11 ± 1.56 ^a^	18.01 ± 1.51 ^b^	19.08 ± 1.68 ^b^
	Total ^2,^**	29.50 ± 1.56 ^a^	21.64 ± 1.38 ^b^	21.47 ± 1.54 ^b^
Syringaldehyde (µg/g)	Free ***	3.07 ± 0.02 ^c^	12.32 ± 0.33 ^a^	10.28 ± 0.36 ^b^
	Bound ***	13.67 ± 0.14 ^c^	18.70 ± 0.64 ^a^	16.33 ± 0.48 ^b^
	Total ^2,^***	16.74 ± 0.16 ^c^	31.02 ± 0.84 ^a^	26.60 ± 0.40 ^b^
Caffeic acid (µg/g)	Free	2.38 ± 0.03 ^a^	2.14 ± 0.18 ^a^	1.99 ± 0.10 ^a^
	Bound	7.87 ± 1.14 ^a^	9.52 ± 1.12 ^a^	10.14 ± 0.44 ^a^
	Total ^2^	10.25 ± 1.17 ^a^	11.65 ± 1.01 ^a^	12.13 ± 0.51 ^a^

^1^ Mean values of three replications; ^2^ Total was calculated as a sum of free and bound values; ^abc^ Values with same alphabet within a row are not significantly different; **, *** Significantly differ at *p *< 0.01, and *p *< 0.001.

**Table 2:** Oxygen radical absorbance capacity (ORAC) and cellular antioxidant activity (CAA) in extracts of mature wheat bran (MWB), immature wheat bran (IWB), and steamed immature wheat bran (SIWB).

Antioxidant Capacity		MWB	IWB	SIWB
ORAC ^1^ (µM TE/g)	Free ***	44 ± 3.9 ^ab^	54 ± 8.3 ^a^	38 ± 3.6 ^b^
	Bound ***	237 ± 4.7 ^b^	273 ± 13 ^a^	252 ± 17 ^ab^
	Total ^2,^***	281 ± 4.7 ^b^	327 ± 11 ^a^	290 ± 20 ^b^
CAA ^1,^*** (µM QE/g)		0.63 ± 0.07 ^c^	4.59 ± 0.17 ^a^	2.58 ± 0.47 ^b^

^1^ Mean values of three replications; ^2^ Total was calculated as a sum of free and bound values; ^abc^ Values with same alphabet within a row are not significantly different; *** Significantly differ at *p *< 0.001.

**Table 3:** EC_50_ values of antiproliferative activity of cells treated with mature, immature, and steamed immature wheat bran extracts.

Bran Extracts	EC_50_ (mg/mL)		
	Caco-2 ***	HT-29 ***	HeLa ***
MWB	17.25 ± 0.84 ^a^	33.25 ± 1.07 ^a^	28.00 ± 2.24 ^a^
IWB	7.62 ± 0.91 ^c^	18.92 ± 2.09 ^c^	7.74 ± 1.31 ^c^
SIWB	15.25 ± 0.18 ^b^	23.43 ± 1.58 ^b^	15.25 ± 0.85 ^b^

Data present the mean of three replicates ± standard error; ^abc^ Values with same alphabet within a column are not significantly different; *** Significantly differ at *p *< 0.001.

**Table 4:** Correlation coefficient (r) between phenolic compositions of mature, immature, and steamed immature wheat bran and antioxidant capacity or cellular antioxidant activity or cell growth inhibition or apoptotic cell death.

Phenolic Compositions		ORAC	CAA	Cell Growth Inhibition			Apoptotic Cell Death
				Caco-2	HT-29	HeLa	
Ferulic acid	Free	−0.170	–0.477	−0.352	−0.191	−0.689 *	−0.278
	Bound	0.815 **	0.988 ***	0.939 ***	0.863 ***	0.975 ***	0.882 ***
Sinapic acid	Free	0.645	0.914 ***	0.829 **	0.786 *	0.927 ***	0.828 ***
	Bound	−0.685 *	−0.530	−0.615	−0.468	−0.356	−0.305
Vanillic acid	Free	0.397	0.054	0.201	0.188	−0.210	0.103
	Bound	−0.428	−0.689 *	−0.581	−0.402	−0.833 ***	−0.471
*p*-Coumaric acid	Free	0.823 **	0.961 ***	0.933 ***	0.748 *	0.933 ***	0.795 ***
	Bound	0.779 *	0.950 ***	0.954 ***	0.918 ***	0.912 ***	0.966 ***
Syringic acid	Free	0.460	0.211	0.297	0.390	−0.085	0.269
	Bound	−0.716 *	−0.871 **	−0.768 *	−0.637	−0.906 ***	−0.619
Syringaldehyde	Free	0.713 *	0.940 ***	0.880 **	0.749 *	0.989 ***	0.812 ***
	Bound	0.781 *	0.961 ***	0.960 ***	0.856 **	0.956 ***	0.890 ***
Caffeic acid	Free	−0.301	−0.488	−0.481	−0.235	−0.675 *	−0.366
	Bound	0.405	0.562	0.446	0.274	0.676 *	0.261

Caco-2, HT-29, and HeLa cells represent relative inhibition % of cell growth for Caco-2, HT-29, and HeLa cells, which were treated with 20 mg/mL of wheat bran extracts. Values in bold with *, ** and *** are different from 0 with significance level at *p *< 0.05, *p *< 0.01 and *p* < 0.001, respectively.
